# Antibacterial Composite Material Based on Polyhydroxybutyrate and Zn-Doped Brushite Cement

**DOI:** 10.3390/polym15092106

**Published:** 2023-04-28

**Authors:** Inna V. Fadeeva, Dina V. Deyneko, Alexander V. Knotko, Anatoly A. Olkhov, Pavel V. Slukin, Galina A. Davydova, Taisiia A. Trubitsyna, Ilya I. Preobrazhenskiy, Alevtina N. Gosteva, Iulian V. Antoniac, Julietta V. Rau

**Affiliations:** 1 A.A. Baikov Institute of Metallurgy and Material Science, Russian Academy of Sciences, Leninsky Prospect 49, 119334 Moscow, Russia; fadeeva_inna@mail.ru; 2 Chemistry Department, Lomonosov Moscow State University, Vorobievy Gory 1, 119991 Moscow, Russia; deynekomsu@gmail.com (D.V.D.); alknt@mail.ru (A.V.K.); 3 Laboratory of Arctic Mineralogy and Material Sciences, Kola Science Centre, Russian Academy of Sciences, 14 Fersman Str., 184209 Apatity, Russia; 4 N.N. Semenov Federal Research Center for Chemical Physics, Russian Academy of Sciences, Kosygina Street 4, Building 1, 119991 Moscow, Russia; aolkhov72@yandex.ru; 5 Plekhanov Russian University of Economics, Stremyanny Lane 36, 117997 Moscow, Russia; 6 State Scientific Center of Applied Microbiology and Biotechnology of Rospotrebnadzor 24, Block A, 142279 Obolensk, Russia; xopgi@yandex.ru; 7 Institute of Theoretical and Experimental Biophysics, Russian Academy of Sciences, Institutskaya 3, 142290 Moscow, Russia; davidova_g@mail.ru (G.A.D.); tta-pro@yandex.ru (T.A.T.); 8 Materials Science Department, Lomonosov Moscow State University, Vorobievy Gory 1, 119991 Moscow, Russia; preo.ilya@yandex.ru; 9 Kola Science Centre RAS, Tananaev Institute of Chemistry, Akademgorodok District 26A, 184209 Apatity, Russia; angosteva@list.ru; 10 Faculty of Materials Science and Engineering, University Politehnica of Bucharest, 313 Splaiul Independentei Street, District 6, 060042 Bucharest, Romania; antoniac.iulian@gmail.com; 11 Academy of Romanian Scientists, 54 Splaiul Independentei Street, District 5, 050094 Bucharest, Romania; 12 Istituto di Struttura della Materia, Consiglio Nazionale delle Ricerche, ISM-CNR, Via del Fosso del Cavaliere 100, 00133 Rome, Italy

**Keywords:** antibacterial, composite material, polyhydroxybutyrate, Zn-doped brushite cement, Zn-substituted brushite cement

## Abstract

A composite material based on electrospinning printed polyhydroxybutyrate fibers impregnated with brushite cement containing Zn substitution was developed for bone implant applications. Powder X-ray Diffraction (PXRD), Fourier Transform Infrared Spectroscopy and Scanning Electron Microscopy were applied for materials characterization. Soaking the composite in Ringer’s solution led to the transformation of brushite into apatite phase, accompanied by the morphology changes of the material. The bending strength of the composite material was measured to be 3.1 ± 0.5 MPa. NCTC mouse fibroblast cells were used to demonstrate by means of the MTT test that the developed material was not cytotoxic. The behavior of the human dental pulp stem cells on the surface of the composite material investigated by the direct contact method was similar to the control. It was found that the developed Zn containing composite material possessed antibacterial properties, as testified by microbiology investigations against bacteria strains of *Escherichia coli* and *Staphylococcus aureus*. Thus, the developed composite material is promising for the treatment of damaged tissues with bacterial infection complications.

## 1. Introduction

Currently, new materials are being requested for bone implants manufacturing due to the exponential increase in the population and, consequently, the number of defects in the bone system due to trauma or aging [1]. There are many osteo-substituting materials, and the most promising of them are biodegradable ones that can be resorbed and replaced by the native bone tissue [2,3]. Osteo-substituting materials must meet a number of requirements, such as an absence of cytotoxicity and satisfactory mechanical compressive strength [4]. Tricalcium phosphate (TCP), due to the similarity of its chemical composition and properties to natural bone tissue, is widely used as a biomaterial for the treatment of bone defects, implant coatings, dental materials, biomedical cements, and other applications [5]. The main advantage of TCP compared to hydroxyapatite (HAP) is a much higher rate of resorption in the body [6]. When using β-TCP as a component of cement powder [7,8,9], a chemical interaction occurs between the components of cement powder and hardening liquid with the formation of dicalcium phosphate dihydrate (DCPD, brushite). Such cements are referred to as brushite cements, according to the main crystalline phase formed as a result of the interaction of cement components.

Previously, we obtained cation-substituted tricalcium phosphates (where the cation was zinc (Zn), manganese, copper, iron or silver) and showed that they exhibit antimicrobial activity [10,11,12,13]. Among them, Zn ions have pronounced antimicrobial activity [14]. Moreover, Zn is an important biological element, playing a role in the growth and development of the body skeleton. It should be also noted that bone tissue contains about 30% of all Zn present in the body, whereas a lack of Zn retards the development of the bone mass. It was also shown that dietary supplements containing Zn have a positive effect on bone metabolism [15].

In addition to its antimicrobial characteristics, an ideal bone replacement material should stimulate the growth of natural bone tissue. For this purpose, it is possible to use growth factors. However, they have a high cost [16] and undesirable side effects [17,18]. Ion supplements are often considered as an alternative to their use; they are not only much cheaper, but also reduce adverse side effects on the body. Moreover, an additional potentially positive effect which can be obtained with the introduction of Zn ions is an increase in the solubility and rate of resorption of TCP, thereby stimulating the formation of the native bone.

One of the disadvantages of brushite calcium phosphate cements (BCPCs), which hampers their use in medicine, is their low strength and crack resistance [19]. Composite materials, including BCPCs and biocompatible polymers, combine the advantages of both components, while the disadvantages of BCPCs are offset by polymer components. Polyhydroxybutyrate (PHB) is a biodegradable polyester which is naturally produced by some microorganisms and is used for energy storage, along with glucose and starch [20,21].

The purpose of this work was to develop a composite material based on PHB and Zn-substituted BCPC (ZnBC), to study its physico-chemical properties, as well as its biocompatibility, cytotoxicity and antibacterial activity. Powder X-ray Diffraction (PXRD), Fourier Transform Infrared Spectroscopy (FT-IR), Scanning Electron Microscopy (SEM) and bending strength investigations were carried out. The NCTC fibroblast cell line from mouse subcutaneous connective tissue was used for the MTT test to investigate the cytotoxicity, while to assess the cell adhesion and spreading, human dental pulp stem cells (DPSC)—easily accessible adult *mesenchymal* stem cells—were applied. Wide-spread Gram-negative *Escherichia coli* (*E. coli*) and Gram-positive *Staphylococcus aureus* (*S. aureus*) bacteria strains were used for the microbiology study. *S. aureus* is known to be the most dangerous among the *Staphylococci* family of bacteria. This pathogen causes a wide variety of infections, common both in community-acquired and hospital-acquired settings.

## 2. Materials and Methods

### 2.1. Synthesis Route

#### 2.1.1. Polymer Preparation

PHB was prepared by electrospinning, as described earlier in [22]. A natural biodegradable polymer—poly-3-hydroxybutyrate series 16F—obtained by microbiological synthesis using BIOMER^®^ (Schwalbach am Taunus, Germany) with a medium-viscosity molecular weight of 2.06 × 105 g/mol, density of 1.248 g/cm^3^, melting temperature peak of 177 °C and a degree of crystallinity of ~60% was used. Ultrathin PHB fibers were obtained by electroforming using a single-capillary laboratory installation EFV-1 (Saint Petersbourg, Russia) at a voltage of 12 kV. Solutions of PHB in chloroform (7 wt.% of PHB) were prepared to obtain fibers.

#### 2.1.2. Cement Preparation

The preparation procedure and characteristics of Zn-substituted brushite cement were described in [23]. Briefly, cement powder was a mixture of Zn-substituted TCP, obtained by precipitation from aqueous solution according to the Equation (1), monocalcium phosphate monohydrate Ca(H_2_PO_4_)_2_·H_2_O (MCPM) and ammonium citrate. An 8% solution of citric acid was used as hardening liquid.

2.5Ca(NO_3_)_2_ + 0.5Zn(NO_3_)_2_ + 2(NH_4_)_2_HPO_4_ + 2NH_3_·H_2_O → Ca_2.5_Zn_0.5_(PO_4_)_2_ +6NH_4_NO_3_ + 2H_2_O (1)

Cement samples were prepared by mixing Zn-substituted β-TCP and MCPM with hardening liquid in a ratio of 3:1 according to the following Equation (2):

Ca_3_(PO_4_)_2_ + Ca(H_2_PO_4_)_2_·H_2_O + 7H_2_O → 4CaHPO_4_·2H_2_O     or Ca_2.5_Zn_0.5_(PO_4_)_2_ + Ca(H_2_PO_4_)_2_·H_2_O + 7H_2_O → 4(Ca,Zn)HPO_4_·2H_2_O (2)

Ammonium citrate interacts with Ca^2+^ ions, forming a poorly soluble calcium citrate on the surface of TCP, which slows down the interaction of the components. Additionally, the introduction of citrate ions should lead to an increase in the cement strength [24].

#### 2.1.3. Composite Preparation

**Figure 1:**
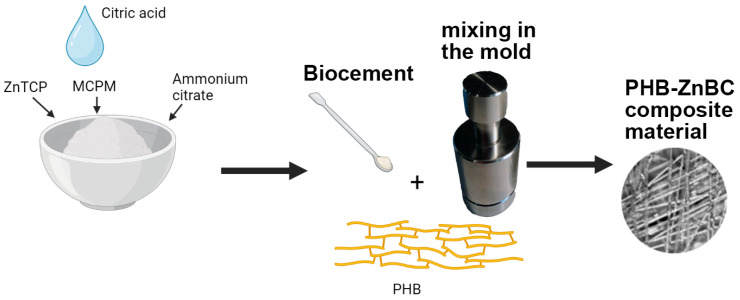
The schematic representation of the preparation procedure of the composite material.

To obtain the composite material, cement paste was applied with a spatula to PHB polymer matrix. Finally, PHG-ZnBC composite material was obtained. The polymer/cement ratio was 1/10. The details on the raw ingredients and synthesized materials are given in Table 1. The schematic illustration of the preparation procedure is shown in Figure 1.

### 2.2. Dissolution Behavior

To simulate the behavior of PHG-ZnBC composite material in the body environment, the samples were immersed in a model Ringer’s solution with a constant temperature of 37 °C for 30 days. Ringer’s solution is a water solution of salts NaCl, KCl, CaCl_2_ with ion concentrations of Na^+^—147 mmol; K^+^—4 mmol; Ca^2+^—2.25 mmol; and Cl^−^—155.6 mmol per 1 L of distillated water. Ringer’s solution is an isotonic solution corresponding to human body fluids. It is commonly used in experiments in vitro to simulate the dissolution processes occurring in native tissues.

### 2.3. PXDR

ZnBC cement and PHB-ZnBC composite material were investigated using the PXRD method. The PXRD patterns were obtained on a Thermo ARL X’TRA powder diffractometer with Bragg–Brentano geometry, Scintillator detector, CuKα radiation, λ = 1.5418 Å (Thermo Fisher Scientific, Waltham, MA, USA). The PXRD data were collected at the 10°–60° 2theta range, with a 0.02° step. The PXRD experiments were performed at room temperature. The phase analysis was carried out by means of the Crystallographica Search-March program (version 2.0.3.1) and the JCPDS PDF#4 database. The PXRD patterns were fitted using Match! Crystal Impact (version 3.14). The crystal structure figures were created using Diamond Software (version 3.2). The Rietveld method was applied for quantitative phase analysis using the JANA2006 software. Crystallographic data including space groups, unit cell parameters and atomic coordinates of β-Ca_3_(PO_4_)_2_ (PDF#4 No 00-009-0169), CaHPO_4_·2H_2_O (PDF#4 No 00-009-0077), Ca_10_(PO_4_)_6_(OH)_2_) (PDF#4 No 00-009-0432) were used as initial parameters. The six-order polynomial was applied for fitting the background and a pseudo-Voigt function for peaks profiles was used. The unit cell parameters were refined, and the atomic coordinates were taken without refinement.

### 2.4. FT-IR Spectroscopy

The IR absorption spectra of the prepared samples were recorded on the Nikolet Avatar-330 infrared Fourier spectrometer (Thermo Fisher Scientific, Waltham, MA, USA), in the range of 4000–400 cm^−1^ with a resolution of 0.9 cm^−1^. The samples were examined in mixtures with potassium bromide (KBr). The IR absorption spectra of PHB and PHB-ZnBC composite material were collected on a Fourier spectrometer FT-803 (Simeks Research and Production Company 2022, Novosibirsk, Russia) in the wavenumber region 4000–400 cm^−1^ with 1 cm^−1^ spectral resolution. The standard KBr disc technique was applied to obtain the spectra.

### 2.5. Bending Strength

The bending strength of cylindrical composite samples was measured, applying the three-point bending method by means of a universal testing machine R-05 (Ivanovo, Russia) equipped with a multi-channel Spider measuring system (Kannapolis, CA, USA). The polymer sample was intimately mixed with brushite cement. This mixture was placed in a cylindrical mold. After cement hardening, the obtained cylinder was cut into pieces: 3 cylindrical composite samples of 5 mm in diameter and of 40 mm in height were prepared, according to the standard. The composite samples were investigated 5 days after their preparation.

### 2.6. Antibacterial Test

The antibacterial activity of the materials was studied against *E. coli* and *S. aureus* bacteria strains. The bacteria were grown in a nutrient medium: Mueller Hinton Agar (HiMedia, Mumbai, India). Three cylindrical composite samples of 5 mm in diameter and 10 mm in height were prepared and used for antibacterial tests. The developed composite samples were first sterilized by the UV radiation (wavelength of 254 nm) for 30 min and then immersed in 0.99 mL of physiological solution (NaCl = 9 g/L aqueous solution) in a 24-well plate. An overnight culture of a strain (10 μL) with a cell concentration of 10^7^ CFU/mL was added to each well. Controls were prepared and tested similarly, but without the PHB-ZnBC composite material.

After 0, 6 and 24 h after the incubation at 37 °C in a thermostat, 0.04 mL of solution was taken from each well and diluted according to decimal method dilution in 0.3 mL of physiological solution. Then, 0.01 mL of suspension was sown from each dilution in a Petri dish containing the nutrient medium and dried for 10 min. After that, bacteria were cultivated in Petri dishes for 24 h at a temperature of 37 °C. Antibacterial activity was determined by estimating the decrease in the CFU level compared to the control sample.

### 2.7. Biocompatibility Tests

The cytotoxicity study of extracts from the powders of the investigated materials was carried out using cells of the NCTC clone L-929 fibroblast cell line of mouse subcutaneous connective tissue by means of the MTT test. The 3-day extracts were prepared in accordance with the requirements of GOST R ISO 10993.12-15 [25].

The adhesion and proliferation of the human Dental Pulp Stem Cells (DPSC) [26] on the surface of the prepared composite material were investigated. Cylinder samples of 8 mm in diameter and 2 mm thickness were placed into the wells of a 24-well plate, after which the DPSC cells were seeded on their surface with a density of 35,000/cm^2^. After 24 and 48 h, the cells were stained with SYTO 9, propidium iodide (both Invitrogen, Thermo Fisher Scientific, Waltham, MA, USA) and Hoechst 33,342 (PanEco, Moscow, Russia). The microphotography of cells was performed using an Axiovert 200 inverted luminescent microscope (Carl Zeiss, Oberkochen, Germany). The cells were counted from images using the ImageJ program.

The fluorescent dye SYTO 9 in the mode of λ_ex_ = 450–490 nm, λ_emiss_ = 515–565 nm stained the DNA and RNA of living and dead cells green, which enabled not only the visualization of cells using a fluorescent microscope, but also the investigation of their adhesion and spreading characteristics on the surface of the material under study. The intercalating reagent propidium iodide (PI) in the mode of λ_ex_ = 546 nm, λ_emiss_ = 575–640 nm stained the nuclei of dead cells red, and in this way it was possible to determine the percentage of non-viable cells.

## 3. Results and Discussion

### 3.1. PXRD Study

The interaction between the components of the ZnBC cement occurred in accordance with Equation (3):

Ca_2.5_Zn_0.5_(PO_4_)_2_ + Ca(H_2_PO_4_)_2_·H_2_O + 7H_2_O → 4(Ca,Zn)HPO_4_·2H_2_O (3)

**Figure 2:**
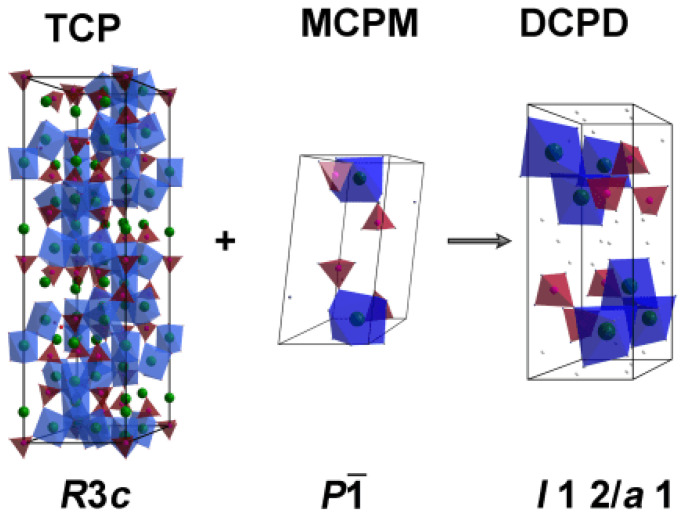
The schematic transformation of the crystal structure of the initial reagents, tricalcium phosphate (TCP) and monocalcium phosphate monohydrate (MCPM), into dicalcium phosphate dihydrate—brushite (DCPD).

The schematic transformation of the initial TCP and MCPM into brushite cement can be represented as shown in Figure 2.

**Figure 3:**
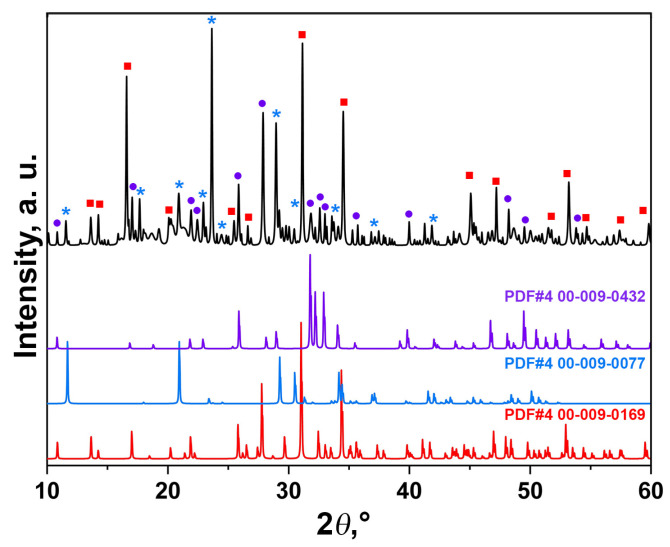
PXRD pattern of the ZnBC cement and the PDF#4 cards (00-009-0169 β-Ca_3_(PO_4_)_2_, 00-009-0077 CaHPO_4_·2H_2_O, 00-009-0432 Ca_10_(PO_4_)_6_(OH)_2_).

The PXRD patterns of the ZnBC cement are shown in Figure 3. The sample consisted of three phases: β-TCP, brushite and HAP, in accordance with the IR spectroscopy data (see below). A slight shift in the diffraction reflections with respect to the PDF#4 card (No. 00-009-0169 β-Ca_3_(PO_4_)_2_) was related to the incorporation of Zn^2+^ ions with smaller ionic radii, compared to Ca^2+^ ions in the β-TCP structure. Thus, according to the Bragg rule, the peaks were shifted towards the higher 2θ° angles (Figure 3). The β-TCP phase was presented in the sample as an initial component, and was the main phase in the investigated cement sample. The formation of the HAP phase occurred due to the transformation of DCPC according to the Equation (4):

10CaHPO_4_·2H_2_O → Ca_10_(PO_4_)_6_(OH)_2_ + 4H_3_PO_4_ + 18H_2_O (4)

The results of the quantitative phase analysis of the ZnBC sample showed that the main phase was β-TCP (64 wt.%). The content of the other phases was as follows: CaHPO_4_·2H_2_O (20 wt.%) and Ca_10_(PO_4_)_6_(OH)_2_ (15 wt.%). We included Ca_2_P_2_O_7_ (1 wt.%) phase in the calculation according to the FT-IR spectroscopy data (see below).

**Figure 4:**
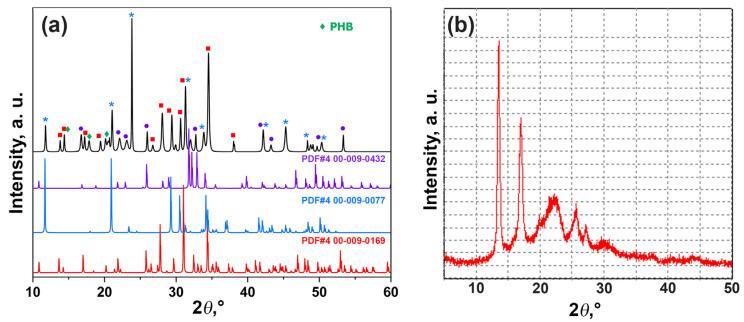
PXRD pattern of the composite material PHB-ZnBC and PDF#4 cards (00-009-0169 β-Ca_3_(PO_4_)_2_, 00-009-0077 CaHPO_4_·2H_2_O, 00-009-0432 Ca_10_(PO_4_)_6_(OH)_2_) (**a**). XRD pattern of the PHB polymer (**b**).

The PXRD patterns of PHB-ZnBC composite material are presented in Figure 4. The sample contained β-TCP, brushite and HAP phases; however, the content of these phases was different with respect to the initial cement sample. The measurements were performed on the sample without grinding. The high intensities of the reflections with indexes (0 4 0) of DCPD (d = 3.800 Å, 2θ = 23.449°), (1 0 2) of HAP (d =3.170 Å, 2θ = 28.198°) and (1 1 0) of TCP (d = 5.210 Å, 2θ = 17.047°), on the PXRD patterns (Figure 3 and Figure 4) were related to the sample texture due to hardening. The reason for the appearance of the texture in the sample is the deviation of the crystallites’ orientation from random [27]. Such a re-distribution of the peak’s intensity is commonly observed in the bone cement materials [28].

The content of PHB in PHB-ZnBC sample was not quantified by the PXRD analysis. The component ratio was PHB:ZnBC = 1:10 (see Materials and Methods section). The results of the quantitative phase analysis for the PHB-ZnBC sample are as follows: Ca_10_(PO_4_)_6_(OH)_2_ (46 wt.%), β-TCP (39 wt.%), CaHPO_4_·2H_2_O (15 wt.%).

### 3.2. Behavior of ZnBC in Ringer Solution

**Figure 5:**
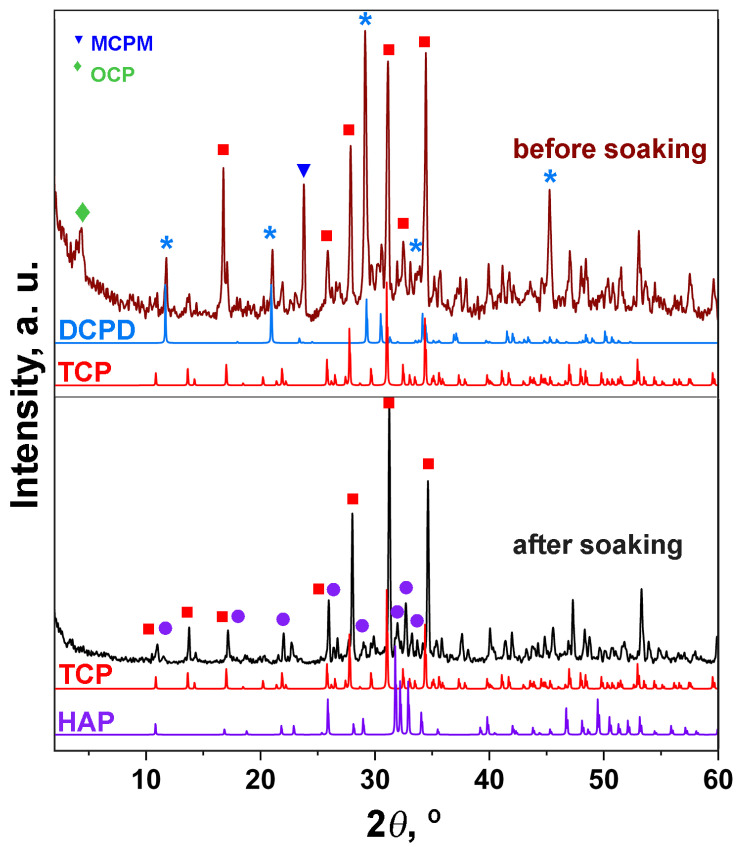
PXRD patterns of ZnBC cement before (**top**) and after (**below**) soaking in the Ringer solution.

The ZnBC cement sample was soaked in the Ringer solution. The PXRD patterns of the sample before (top) and after (bottom) soaking are shown in Figure 5. The initial sample contained the impurities of the MCPM (as raw material) and the octacalcium phosphate (Ca_8_(HPO_4_)_2_(PO_4_)_4_·5H_2_O, OCP), which were formed according to the Equations (5) and (6):

10CaHPO_4_·2H_2_O + H_2_O → Ca_8_(HPO_4_)_2_(PO_4_)_4_·5H_2_O (5)

3Ca_3_(PO_4_)_2_ + 7H_2_O → Ca_8_(HPO_4_)_2_(PO_4_)_4_·5H_2_O + Ca(OH)_2_ (6)

The Ca(OH)_2_ phase was not present in the PXRD pattern, since it immediately reacted with H_3_PO_4_ (from the reaction (3)) with the formation of the TCP phase according to the Equation (7):

3Ca(OH)_2_ + 2H_3_PO_4_ → Ca_3_(PO_4_)_2_ + 6H_2_O (7)

After soaking, the main phase was β-TCP with the impurity of HAP (Figure 5, bottom). According to the quantitative phase analysis, the content of β-TCP was 76 wt.%, while HAP was 24 wt.%. The formation of the HAP-type phase took place according to the following equations:

(1) The most unstable OCP → HAP
  Ca_8_(HPO_4_)_2_(PO_4_)_4_·5H_2_O → 4Ca_10_(PO_4_)_6_(OH)_2_ + 6H_3_PO_4_ + 17H_2_O (8)
  or
  Ca_8_(HPO_4_)_2_(PO_4_)_4_·5H_2_O → ½ Ca_10_(PO_4_)_6_(OH)_2_ + 3CaHPO_4_ (9)
(2) DCPD → HAP
  10CaHPO_4_·2H_2_O → Ca_10_(PO_4_)_6_(OH)_2_ + 4H_3_PO_4_ + 18H_2_O (10)
(3) TCP → HAP
  10Ca_3_(PO_4_)_2_ + 6H_2_O → 3Ca_10_(PO_4_)_6_(OH)_2_ + 2H_3_PO_4_ (11)

According to the PXRD data, it follows that brushite cement is characterized by a continuous transformation of the calcium phosphate salts.

### 3.3. FT-IR Study

**Figure 6:**
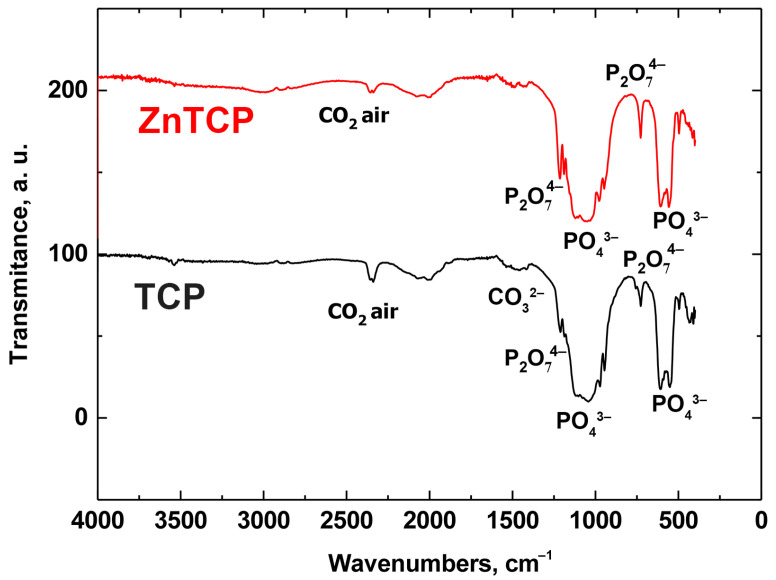
FT-IR spectra of powders of β-TCP (black line) and ZnTCP (red line).

The FT-IR spectra of powders based on β-TCP and Zn-substituted β-TCP (ZnTCP) (Figure 6) show the regions of the most intense oscillations, corresponding to the PO_4_^3−^ (ν4: 565, 603 cm^−1^; the region at 900–1200 cm^−1^ [29]). The intense oscillations attributed to the P_2_O_7_^4−^ group with valence oscillations of the P–O–P bond were registered. The appearance of pyrophosphate groups may be associated with the thermal decomposition of brushite according to the Equation (12):

2CaHPO_4_·2H_2_O →Ca_2_P_2_O_7_ + 5H_2_O↑ (12)

**Figure 7:**
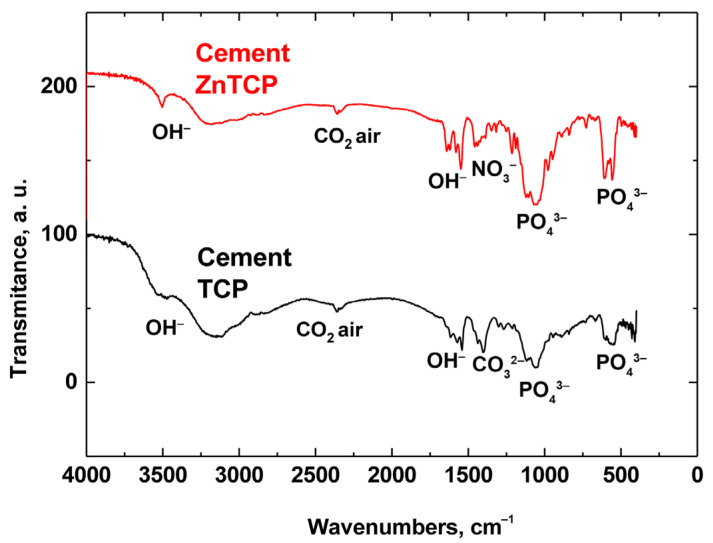
FT-IR spectra of pure BC and ZnBC cements.

FT-IR spectra of ZnBC cement, and pure BC cement for comparison, are shown in Figure 7. It can be observed that in brushite cement there was an intense peak at 3570 cm^−1^, as well as a peak at 632 cm^−1^, which corresponds to the deformation vibrations of the OH^−^ group [30]. In addition, there were reflections attributed to the CO_3_^2−^ groups at 1300–1550 cm^−1^ [31]. Additionally, at 2350 cm^−1^, peaks attributable to CO_2_ from the air [32] were detected. The peaks attributed to the nitrate residues from the raw materials were registered at 1380 cm^−1^.

**Figure 8:**
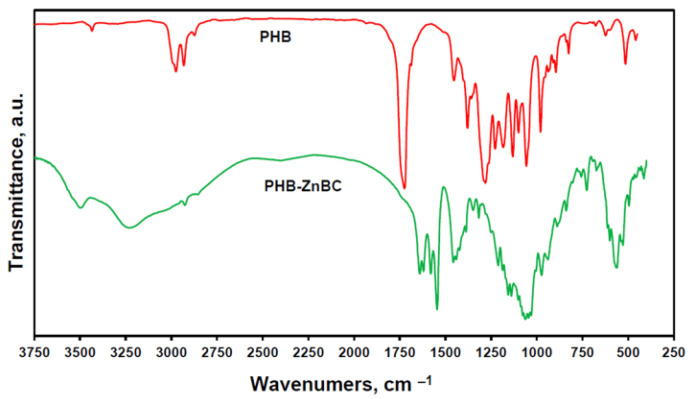
FT-IR spectra of PHB polymer and PHG-ZnBC composite material.

The IR spectra of pure PHB polymer and PHG-ZnBC composite material are shown in Figure 8. Characteristic bands of all groups of atoms included in the structure of the samples can be observed. The absorption bands in the region of 3500–3220 cm^−1^ refer to the stretching vibrations of OH^−^, and the band at 615 cm^−1^ refers to the bending vibrations of OH^−^ [33,34]. The vibrations of the functional groups of the PHB polymer were registered in both the samples. These vibrations were attributed to the CH– group peaks at 3000–2800 and 1450–1200 cm^−1^. Additionally, there was a large number of stretching vibrations of C–O, C–O–C and C–C–O groups in the range of 935–1200 cm^−1^, while bending vibrations were detected at 500–700 cm^−1^ [35]. Carbonyl stretching peaks at 1620–1720 cm^−1^ were also present. The phosphate ions present in ZnBC were detected as orthophosphate (572–1137 cm^−1^) and pyrophosphate (730 cm^−1^) [36,37]. All the detected bands are summarized in Table 2.

### 3.4. SEM Observations

**Figure 9:**
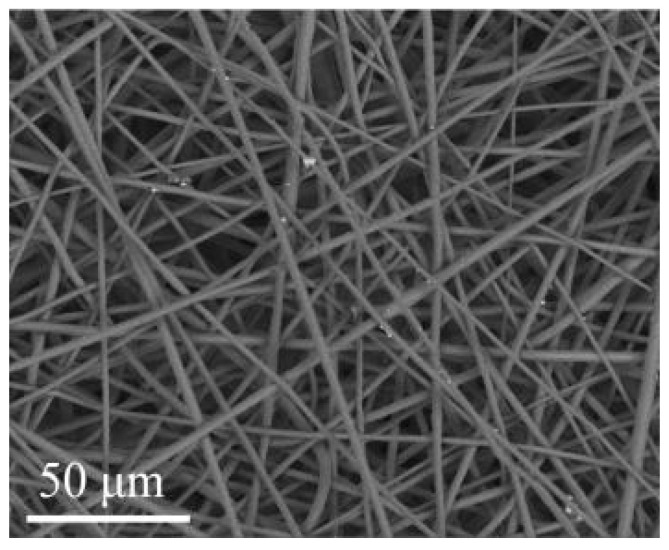
SEM image of PHB polymer obtained by electrospinning.

An SEM image of the PHB polymer obtained by electrospinning is shown in Figure 9. It consists of randomly distributed fibers of a regular cylindrical shape with an average diameter of 2–5 microns.

**Figure 10:**
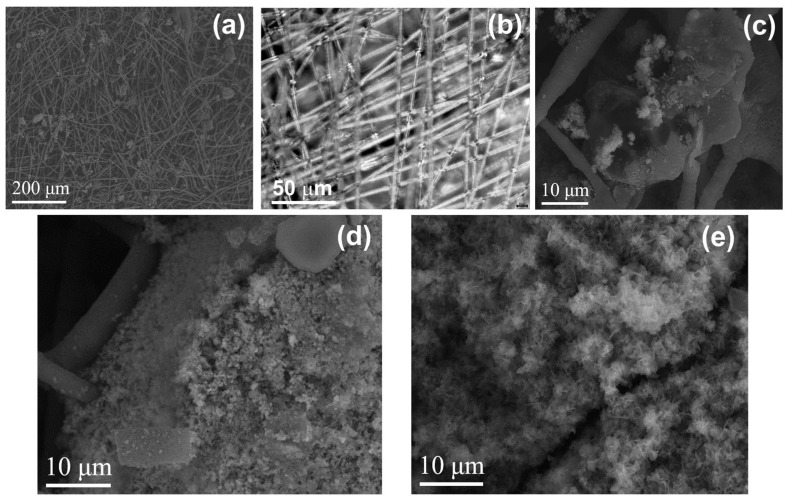
SEM images of the composite PHB-ZnBC at different magnifications (**a**–**c**). SEM images of the composite PHB-ZnBC: 5 days after the sample preparation (**d**); after soaking for 30 days at 37 °C (**e**).

The microstructure of the PHG-ZnBC composite material (Figure 10a–c) was heterogeneous, with the crystalline phase of the ZnBC cement irregularly distributed over the PHB fibers. After soaking the composite in the Ringer solution at 37 °C for 30 days, the changes in the phase composition occurred, as discussed above in Section 3.2 and shown in Figure 5. These transformations were accompanied by changes in the microstructure of the composite (Figure 10d,e). As a result of soaking, the size of the cement crystals increased and their shape became lamellar, in accordance with the results reported in [23]. The appearance of lamellar crystals on the surface of the sample (Figure 10e) was related to the formation of the HAP phase, in agreement with the obtained PXRD results (Figure 5). This type of particle shape is characteristic for the apatite phases [38].

### 3.5. Bending Strength Measurements

**Figure 11:**
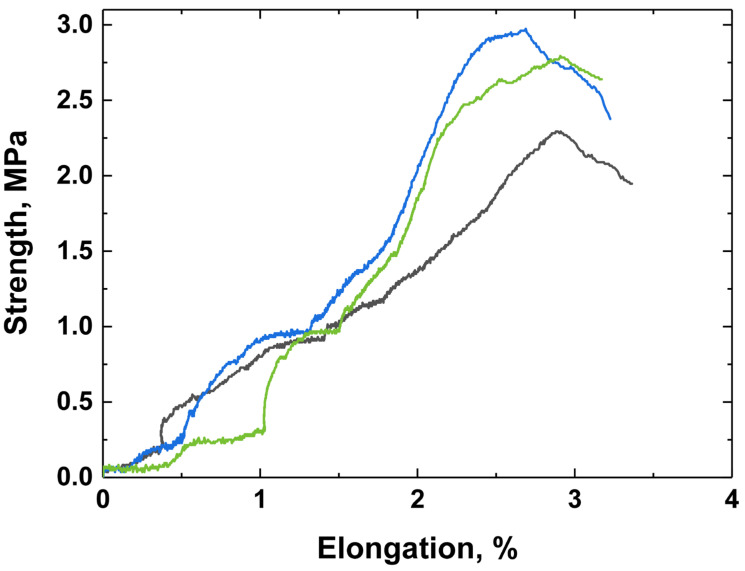
Bending strength of PHB-ZnBC composite material 5 days after preparation.

The strength of the PHB-ZnBC composite material was measured according to the three-point bending strength method 5 days after the sample preparation, and the obtained experimental data are demonstrated in Figure 11. The bending strength of the composite material was determined to be 3.1 ± 0.5 MPa, which meets the requirements for biomaterials for bone treatment [39].

According to the literature data, the compressive strength of brushite cements is 10–15 MPa [40]. This fact is related to poor crystal compaction during hardening due to a fast setting time. In our previous work, the compressive strength of Zn-containing brushite cement was determined to be 17.5 ± 1.6 MPa [23].

The bending strength of brushite cements was relatively low and did not exceed 4.5 MPa [41]. The presence of β-TCP in the cement can improve this value, acting as filler particles [42]. The flexural strength of pure PHB according to [43] is about 60 MPa.

In the present manuscript, the bending strength of the PHB-ZnBC composite was measured to be 3.1 ± 0.5 MPa. This value was related to a low crystal compaction of the composite material because of the texturing of the sample. The texture appeared due to the orientation of the PHB fibers being predominantly in one plane (2D structure), as can be observed from the SEM images (Figure 9 and Figure 10). The presence of PHB did not significantly affect the bending strength of the composite due to its low content (PHB:ZnBC = 1:10).

### 3.6. Antibacterial Activity

**Figure 12:**
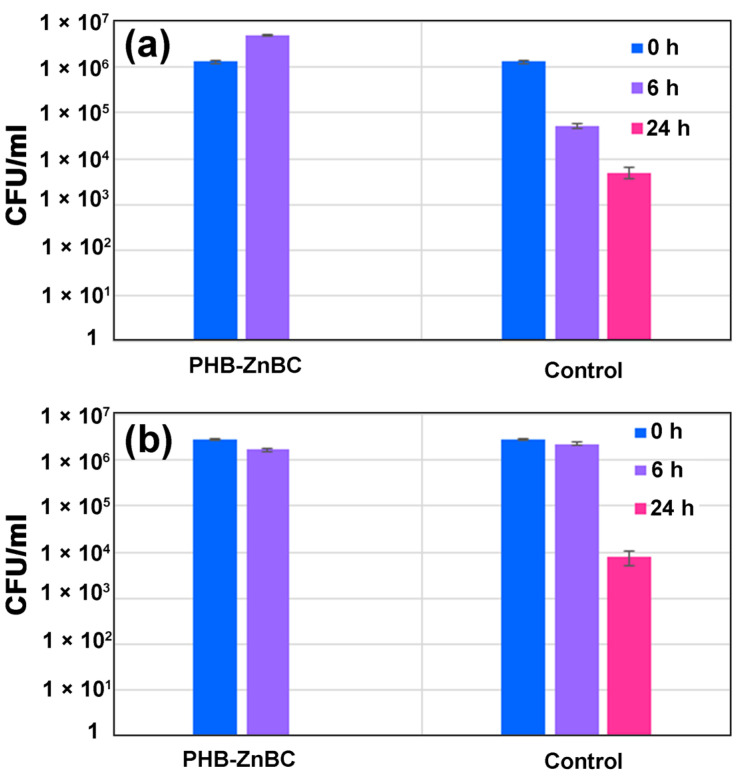
Strain CFU concentration levels of *E. coli* (**a**) and *S. aureus* (**b**) after incubation with PHB-ZnBC sample and in the control.

The antibacterial activity of the developed PHB-ZnBC composite material was tested against *E. coli* and *S. aureus*, applying the agar overlay method [44]. The data on the bacteria growth inhibition are presented in Table 3 and Figure 12. In the present research, bacteria were cultivated in a nutrient-free physiological solution to assess the contribution of the antibacterial effect of our composite material without the contribution of culture growth. The direct contact with the material simulated the physiological conditions: the composite polymer–cement material is set directly into the human body and comes into direct contact with antibiotic-resistant hospital bacteria, such as *E. coli* or *S. aureus*. A similar experimental approach was previously described in [45,46].

As can be observed from Table 3 and Figure 12, at the beginning of the test, different results were obtained for the applied bacteria strains. The concentration of *E. coli* slightly increased after 6 h from (1 ± 0.01) × 10^6^ to (5 ± 0.01) × 10^6^ CFU/mL, compared to the control, in which the *E. coli* content, in contrast, decreased by an order of magnitude (to (5 ± 0.01) × 10^4^ CFU/mL). Under the same conditions, in the case of *S. aureus*, the bacteria number decreased slightly after 6 h (from (3 ± 0.01) × 10^6^ to (2 ± 0.01) × 10^6^ CFU/mL), similar to the control. However, no viable *E. coli* and *S. aureus* bacteria were detected in either of the experiments after 24 h (in the control, the numbers of both bacteria species after 24 h were (6 ± 0.05) × 10^3^ and (1 ± 0.05) × 10^4^ CFU/mL, respectively). Thus, the developed PHB-ZnBC composite had a significant antibacterial effect, which developed within 24 h.

However, during the first 6 h, the PHB-ZnBC sample had no antibacterial effect, likely due to the low solubility of the composite. The investigated sample based on brushite cement consisted of several phases, according to the PXRD study. Despite the continuous phase transformations of the cement material, all phases belonged to calcium phosphates (Figure 5). According to quantitative phase analysis, the main phase was Ca_10_(PO_4_)_6_(OH)_2_ (46 wt.%), characterized by a relatively slow dissolution rate; β-TCP was the second phase (39 wt.%), characterized by a higher solubility with respect to HAP. This is likely the reason that the bactericidal concentration of Ca^2+^ and Zn^2+^ ions was reached only after 18–24 h. A similar time for the inhibition of bacterial growth was observed in [47]. The PHB polymer alone did not show any antibacterial effect, according to the literature reference [48]. For this reason, the complete inhibition of *E. coli* or *S. aureus* growth was attributed to the ZnBC cement material.

### 3.7. The Viability of Cells on the Composite Material

**Figure 13:**
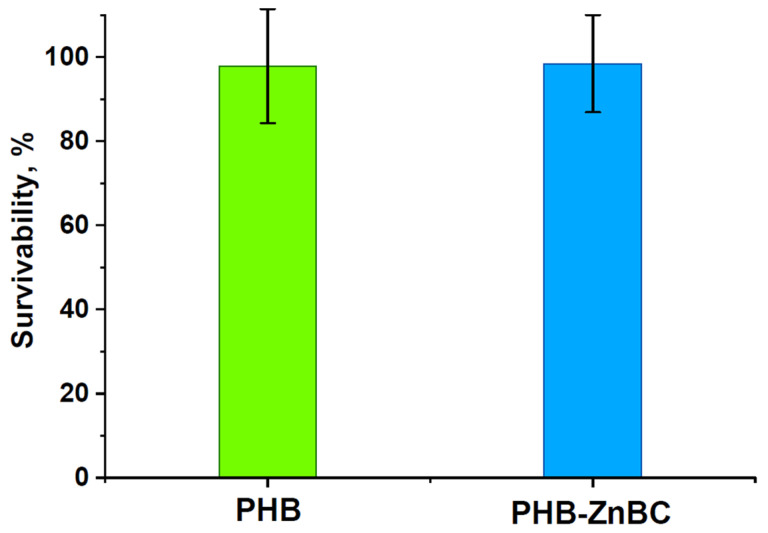
MTT test results of the metabolic activity of the NCTC cells for 24 h incubation with 3-day extracts. Control sample—glass slide—corresponds to 100%. The error bar is the mean SD.

The results of the MTT test regarding the metabolic activity of the NCTC cells for 24 h of incubation with 3 day extracts are shown in Figure 13. The glass slide was used as the control sample; it corresponded to 100%. The error bars on the column graphs corresponded to the mean standard deviation (SD). The results of the cytotoxicity study demonstrated that the extracts from the developed materials, PHB and PHB-ZnBC, did not significantly influence the viability of the NCTC mouse fibroblast cells, the cell survival being 97% and 98%, respectively, with respect to the control, and therefore the tested materials were not cytotoxic.

**Figure 14:**
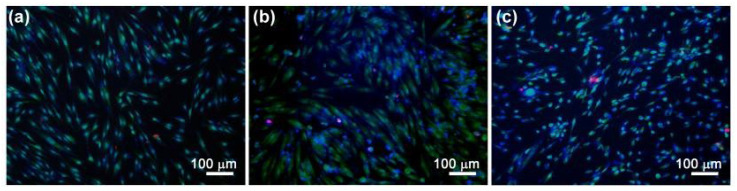
DPSC cells cultured for 48 h on the surface of the samples: PHB (**a**), PHB-ZnBC (**b**), control (**c**). Color: SYTO 9 (green), Hoechst 33258 (blue), PI (red).

**Figure 15:**
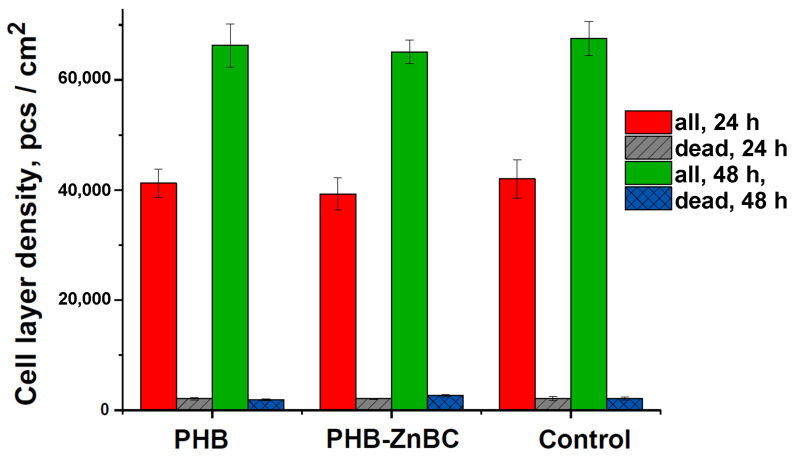
The density of the cell layer and the number of dead DPSC cells on the studied samples after 24 and 48 h of cultivation.

The DPSCs viability was assessed by the differentiated fluorescent staining of living and dead cells using fluorescent dyes. Images of cells stained with SYTO 9 (stains the nuclei and cytoplasm of all cells green (λ_ex_ = 450–490 nm, λ_emiss_ = 515–565 nm)), propidium iodide (stains the nuclei of dead cells red (λ_ex_ = 546 nm, λ_em_ = 575–640 nm)) and Hoechst 33,342 (stains in blue the nuclei of all cells (λ_ex_ = 343 nm, λ_em_ = 483 nm)) are presented in Figure 14, and the data on the number of cells in Figure 15.

As can be observed from Figure 14, there was a large number of cells on the surface of the samples, the cells were homogeneously distributed and spread, and they were characterized by a normal morphology. The number of dead cells was insignificant (see Figure 14 and Figure 15).

Moreover, there were no significant differences in the number of cells on all the studied materials, as shown in Figure 15, in which the DPSC density layer is presented. As can also be observed from Figure 15, the cell density layer for all the samples was significantly increased after 48 h of cultivation, with respect to 24 h.

## 4. Conclusions

In this work, a composite material based on PHB, printed by electrospinning, and ZnBC cement was developed. According to PXRD and FT-IR investigations, the ZnBC cement and the composite PHB-ZnBC sample consisted of several calcium phosphate phases. After soaking the composite in the Ringer solution at 37 °C for 30 days, the transformation of the ZnBC cement material into mixture of β-TCP and HAP phases took place. This transformation was accompanied by changes in the microstructure; the size of cement crystals increased and their shape changed to lamellar. The bending strength of the composite was found to be 3.1 ± 0.5 MPa.

The investigation of the metabolic activity of the NCTC mouse fibroblast cells with the extract from the developed composite material testified that it did not noticeably affect the cell viability. The direct contact method, applied to study the adhesion and spreading of the human DPSC cells on the surface of the composite sample, showed that the morphology and the number of cells was similar to the control.

The composite PHB-ZnBC material possesses antibacterial characteristics and showed a complete inhibition of bacterial growth after 24 h of incubation for both *E. coli* and *S. aureus*.

The developed composite material is promising for bone replacements that are prone to infection.

## Figures and Tables

**Table 1:** Information about raw materials, synthesized zinc-containing brushite cement, and composite material.

Cement Components	Chemical Formula	Properties
ZnTCP	Ca_2.5_Zn_0.5_(PO_4_)_2_	200–400 μm
MCPM	Ca(H_2_PO_4_)_2_·H_2_O	200–400 μm
Ammonia citrate	HOC(CO_2_H)(CH_2_CO_2_NH_4_)_2_	200–400 μm
Citric acid	HOC(CO_2_H)(CH_2_CO_2_H)_2_	Water solution, 8 wt.%
ZnBC	(Ca,Zn)HPO_4_·2H_2_O	Setting time—4–5 min, Hardening time—24 h
PHB-ZnBC	PHB-(Ca,Zn)HPO_4_·2H_2_O	Materials ratio PHB:ZnBC = 1:10

**Table 2:** Vibration modes in the FT-IR spectra of PHB polymer and PHB-ZnBC composite.

Assignment	IR Peaks PHB, cm^−1^	IR Peaks PHB-ZnBC, cm^−1^
ν_as_[OH^−^] + ν_s_[OH^−^]	3441	3219, 3481
ν_as_[CH_2_]	2928, 2982	2918
ν_s_[CH_2_]	2882	2861
ν[RCO–O]	1690, 1720	1623, 1637
ν[C–O]	-	1548, 1582
δ[CH_2_]	1458	1419, 1437, 1453
ω[CH_2_]	1365, 1377	1315, 1344, 1386
τ[CH_2_]	1227, 1288	1236
ν_s_[P–O–P] in P_2_O_7_^4−^	-	1208
ν_as_[C–O–C]	1103, 1130, 1180	1104, *1137*, 1155, 1180
ν_3_[PO_4_^3−^]	-	*1137*
ν[C–C] + ν[C–O]	1057	1012, 1040, 1056, 1068, 1073, 1084, 1090
ν_3_[PO_4_^3−^]	-	*1040*, *1056*, *1068*, *1073*, *1084*, *1090*
νs[C–O–C] + ν[C–C] + ρ[CH_2_]	910, 940, 953, 980	935, 977
ν_1_[PO_4_^3−^]	-	*977*
ρ[C–H_2_] + ν[C–O]	825, 837, 871, 895	800, 836, 883
ν_s_[P–O–P] in P_2_O_7_^4−^	-	730, *753*
δ[C–C–O] + δ[C–O–C]	606, 671, 687	600, 670, 697
δ[OH^−^]	629	615
δ[C–O–C] + δ[C–C–O]	459, 513	572
ν_4_[PO_4_^3−^]	-	*572*

Note: The intervals of the characteristic bands for the [PO_4_^3−^] groups and the PHB bands [C–O–C], [C–C–O], [C–C] and [C–O] are overlapped with each other. Therefore, the repeating frequencies are in italics.

**Table 3:** Strain CFU concentration levels of *E. coli* and *S. aureus* during incubation with PHB-ZnBC composite and in the control after 0, 6 and 24 h.

Bacteria	Sample	0 h	6 h	24 h
*E. coli*	PHB-ZnBC	1 ± 0.01 × 10^6^	5 ± 0.01 × 10^6^	0
	Control		5 ± 0.01 × 10^4^	6 ± 0.05 × 10^3^
*S aureus*	PHB-ZnBC	3 ± 0.01 × 10^6^	2 ± 0.01 × 10^6^	0
	Control		2 ± 0.01 × 10^6^	1 ± 0.05 × 10^4^

## Data Availability

The research data are available upon an official reasonable request.
